# Anticipated Affect That Encourages or Discourages Human Papillomavirus Vaccination: A Scoping Review

**DOI:** 10.3390/vaccines11010124

**Published:** 2023-01-04

**Authors:** Tsuyoshi Okuhara, Marina Terada, Yumi Kagawa, Hiroko Okada, Takahiro Kiuchi

**Affiliations:** Department of Health Communication, School of Public Health, The University of Tokyo, Tokyo 113-8655, Japan

**Keywords:** human papillomavirus, HPV vaccination, anticipated affect, anticipated regret, emotion, health communication

## Abstract

We reviewed studies that examined the anticipated affects associated with human papillomavirus (HPV) vaccination to identify gaps in the literature and the currently available practice implications for encouraging HPV vaccination. We systematically searched MEDLINE, the Cumulative Index to Nursing and Allied Health Literature, PsycINFO, PsycArticles, Academic Search Complete, Scopus, and Web of Science to find English articles that quantitatively and qualitatively examined anticipated affects associated with HPV vaccination. A total of twenty-one studies were identified. Seventeen studies examined the anticipated inaction regret (i.e., not being vaccinated). Most of the included studies reported that anticipated inaction regret had a significantly positive association with HPV vaccination outcomes, such as vaccination behavior, intention, willingness, and acceptability. Furthermore, seven studies reported that anticipated inaction regret had a significantly positive and stronger association with vaccination outcomes than cognitive beliefs, such as vaccine effectiveness and safety, and perceived susceptibility and severity. The present review indicated that the stronger the participants’ anticipated inaction regret, the more likely they were to receive the HPV vaccine. Messages targeting the anticipated affect may be as effective as or more effective than messages targeting cognitive beliefs in encouraging HPV vaccination among people. However, most of the studies included in the present review adopted a cross-sectional design with vaccination intention and willingness as outcomes. Therefore, future studies should examine the influence of anticipated affects on the utilization of HPV vaccines using experimental designs to accumulate stronger evidence.

## 1. Introduction

Cervical cancer is the fourth most common cancer affecting females, with more than 570,000 new cases reported annually, and approximately 311,000 females dying of the disease annually worldwide [1]. Cervical cancer is a preventable disease. Several preventive strategies are available, with human papillomavirus (HPV) vaccination as the most common method. In November 2020, the World Health Organization announced the “Global Strategy to Accelerate the Elimination of Cervical Cancer as a Public Health Problem” and suggested vaccinating 90% of females against multiple strains of HPV at the age of 15 years [1]. HPV vaccine coverage tends to be higher in high-income countries; however, the coverage remains low in some high-income countries, such as Japan (1%), Italy (27%), France (33%), Germany (43%), and the United States (49%), including girls aged 15 years who received the recommended doses of HPV vaccine in 2019 [2]. A recent study reported that the final dose of HPV vaccine coverage in 2019 for females remained at 40% only in high-income countries and was much lower in other countries [3]. Hence, government agencies and healthcare professionals should continue to communicate with individuals and communities to encourage HPV vaccination.

Previous research and practices on communication to encourage vaccination have primarily adopted cognitive-behavioral models and targeted cognitive beliefs, such as the perceived susceptibility and severity of infection [4,5]. Previous studies on HPV vaccination promotion also adopted behavioral models, such as the health belief model and theory of planned behavior, with a focus on cognitive beliefs, such as perceived susceptibility to infection, the seriousness of the disease, and vaccine effectiveness and safety [6,7]. Previous studies using these cognitive-behavioral models assumed that an individual’s decisions are logical and rational and that an individual’s cognitive beliefs about vaccination can be used to predict their vaccination status in the future [8]. Existing cognitive-behavioral models, however, have been criticized for focusing solely on the influences of cognitive beliefs on health behaviors and failing to consider the affective influences [9].

Recent studies have examined the affective determinants of health behaviors as a complement to cognitive-behavioral models [10]. Anticipated affect, which is the expectation of an affective response to the target behavior (e.g., “If I do not receive the HPV vaccine, I will feel regret”), has received much attention as an affective determinant of health behavior. Anticipated affect is distinct from other concepts of affect such as core affect (such as hedonic responses [pleasure or displeasure] and arousal), emotions that are currently experienced (such as fear, anger, etc.), and moods (such as depression) [10]. Studies on anticipated affect focus on the affect that is expected to occur after a performance or nonperformance of a behavior rather than the affect that is expected to occur while the behavior is being performed [9]. Additionally, studies on anticipated affect focus on self-conscious emotions (such as regret, guilt, worry, etc.) rather than hedonic emotions (such as enjoyment, excitement, etc.) which have been the focus in studies that measured experiential attitudes [9].

The majority of the studies investigated the anticipated inaction negative affects, such as inaction regret and, to a lesser extent, inaction guilt [11]. For example, if individuals do not receive HPV vaccination, they will anticipate feeling regretful if they develop the infection in the future; thus, they opt to receive HPV vaccination just to avoid feeling such negative affects. Anticipated inaction regret was associated with engagement in health behaviors, such as physical activity [12], cancer screening [13,14], and vaccination [15]. Additionally, anticipated inaction regret was a stronger predictor of vaccinations [16,17] than cognitive beliefs such as perceived susceptibility and severity. By contrast, anticipated action negative affect (such as an anticipated action regret) may discourage some individuals from receiving HPV vaccination. For example, some individuals may not receive the HPV vaccination because they expect to regret it if they experience severe vaccine adverse events.

Thus, anticipated affects may be positively or negatively associated with and influence HPV vaccination. An overview of previous studies on how anticipated affects relate to and influence HPV vaccination would contribute to the development of effective communication strategies to encourage HPV vaccination. This review aimed to provide an overview of the studies investigating the anticipated affects that aimed to encourage HPV vaccination. This review also aimed to explore the usefulness of affective influence and compare it with the cognitive influence of HPV vaccination. The following research questions were raised:

RQ1: What is the state of the art of previous studies on the anticipated affects associated with HPV vaccination (e.g., type of anticipated affect, study design, participant characteristics, and main findings)?

RQ2: What gaps exist in previous studies on the anticipated affects associated with HPV vaccination, and what studies are needed to fill in these gaps?

RQ3: What types of anticipated affects (in comparison with cognitive beliefs) relate to and influence the uptake of HPV vaccination?

RQ4: Is there any evidence-based recommendation on communication that focuses on anticipated affects to encourage HPV vaccination?

## 2. Materials and Methods

The current review was conducted in accordance with the preferred reporting items for systematic reviews and meta-analysis extensions for scoping reviews [18] (Appendix A).

### 2.1. Literature Search

A literature search was conducted in several databases using the EBSCOhost search platform: MEDLINE, Cumulative Index to Nursing and Allied Health Literature, PsycINFO, PsycArticles, and Academic Search Complete. The Scopus and the Web of Science platforms were also searched. The database searches were conducted by the first author (TO) on 18 July 2022, and only articles published in or after 2000 were considered eligible for analysis. The following combinations of keywords were used to search for abstracts: (anticipated OR anticipatory) AND (affect OR affective OR emotion OR regret OR guilt OR worry OR fear OR disgust OR embarrassment OR pride OR satisfaction) AND (vaccines OR vaccinations OR immunizations OR vaccine hesitancy OR vaccine refusal OR vaccine reluctance OR vaccine confidence OR vaccine willingness OR vaccine acceptance OR vaccination hesitancy OR vaccination refusal OR vaccination reluctance OR vaccination confidence OR vaccination willingness OR vaccination acceptance). All search results were imported into Rayyan QCRI software to ensure a systematic and comprehensive search and to document the selection process [19]. The reference lists of the eligible studies were also screened to identify potentially eligible studies. Google Scholar was used to triangulate the selection of studies.

### 2.2. Eligibility Criteria

The present study included articles that quantitatively or qualitatively examined the anticipated affects and aimed to encourage HPV vaccination. Studies of other types of vaccines, such as those against coronavirus disease 2019, influenza, measles, mumps, and rubella, were excluded. Studies on other types of vaccines, such as HPV, influenza, and measles-mumps-rubella, were excluded. Studies on other concepts of affect than anticipated affect, such as core affect (such as hedonic responses [pleasure or displeasure] and arousal), emotions that are currently experienced (such as fear), and moods (such as depression), were excluded. Studies on any type of anticipated affect were eligible. Any type of study was eligible, including quantitative (e.g., intervention, longitudinal, and cross-sectional), qualitative, and review studies. Studies that quantitatively assessed the outcomes, including behavior, behavioral intention, and attitude, were eligible. Studies on participants of any age, sex, ethnicity, or country were also eligible. Only papers published in English were included in this study. Studies not published in the full text were excluded. Gray literature (such as conference proceedings, theses, and dissertations) was included if sufficient information was provided to confirm its eligibility.

### 2.3. Study Selection

**Figure 1:**
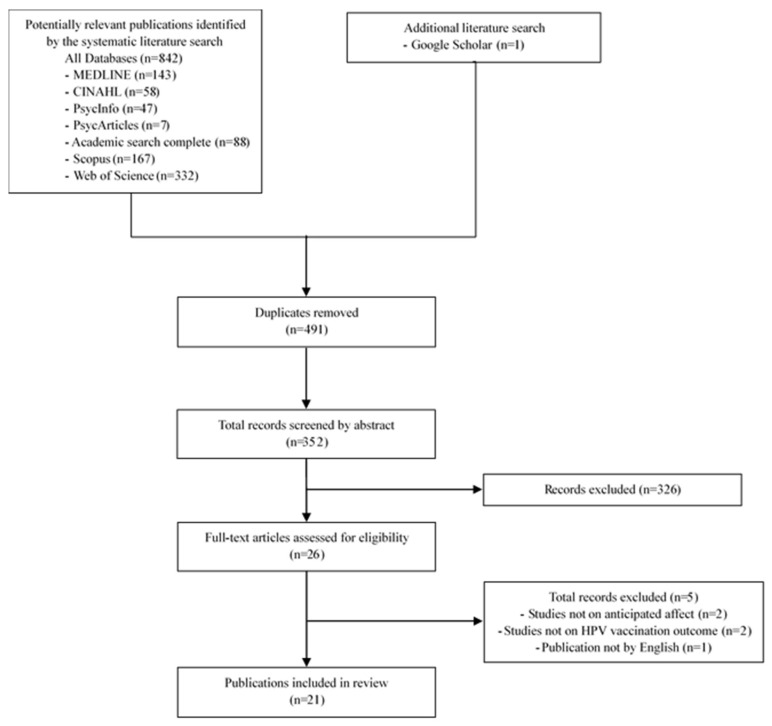
Search process flow chart.

Study selection was performed using Rayyan QCRI software [19]. Two independent reviewers (the first and third authors, TO and YK, respectively) screened the titles and abstracts of all studies that met the eligibility criteria. Disagreements were resolved by discussion until a consensus was reached, and the opinion of a third reviewer (the fourth author, HO) was sought when necessary. Full-text versions of potentially relevant studies were retrieved and screened independently by two reviewers (the first and second authors, TO and MT, respectively). Disagreements were resolved by discussion until a consensus was reached, and the opinion of a third reviewer (the fourth author, HO) was sought when necessary. Figure 1 shows the results of the literature search and study selection process.

### 2.4. Data Extraction and Synthesis

A custom data extraction form was created to obtain all relevant data from each study. The data extraction form was piloted with a sample of eligible studies to assess its reliability in extracting the targeted study data. The first author (TO) conducted the data extraction, whereas the second author (MT) checked the extracted data against the full texts of the studies to ensure that no omissions or errors were committed. Disagreements were resolved through discussion until a consensus was reached. The extracted data were as follows: publication type, study characteristics (author, year of publication, and country), study aim, study design, type of anticipated affect (such as regret and worry), type of cognitive beliefs (such as perceived susceptibility, severity of infection, and vaccine effectiveness [when examined]), study setting (such as internet, classroom, clinic), participants’ characteristics (such as number, sex, and age), methodology (such as methods for interviewing and outcomes), main results and findings, and antecedents and mediating factors (when examined). A numerical summary was used to describe the characteristics of the included studies. The findings were summarized in tables and synthesized using descriptive narrative reviews.

## 3. Results

### 3.1. Study Characteristics

A total of 21 studies were included in this review (Table 1). One study was published in 2000, seventeen in 2010, and three in 2020. Twelve studies were conducted in the United States, two in Hong Kong, two in Romania, one in the United Kingdom, one in the Netherlands, one in Italy, one in Canada, and one in Korea. Nineteen studies examined anticipated inaction regret, nine examined anticipated action regret, one examined anticipated inaction regret and worry, two examined anticipated inaction worry, one examined anticipated anxiety reduction, and one examined anticipated positive and negative affect. Two studies were qualitative, whereas nineteen were quantitative. Among the quantitative studies, thirteen adopted a cross-sectional design, three adopted a longitudinal design, and three were intervention studies that adopted the between-subject design with random allocation. Parents of adolescent girls were included in eight studies; parents of adolescent males in two studies; mothers of adolescent boys in one study; undergraduate students in two studies; college-aged males in one study; young adults in three studies; heterosexual males in one study; gay and bisexual adults in one study; and lesbian and bisexual young females in one study. The minimum number of participants was 219, whereas the maximum number of participants was 979 (median: 368 in quantitative studies). With regard to the primary outcome of quantitative studies, nine studies measured vaccination intention, six measured self-reported vaccination behavior, two measured vaccine acceptability, and two measured vaccination willingness. All studies with cross-sectional and longitudinal designs examined the psychosocial cognitive variables such as the perceived severity of infection and vaccine effectiveness, social norms, and anticipated affect.

### 3.2. Summary of Key Findings

Table 2 summarizes the key findings based on the type of anticipated affect and the primary outcome. Five studies used a cross-sectional design to examine the association between anticipated inaction regret (i.e., not being vaccinated) and vaccination intention [21,32,33,37,39]. All five studies reported that anticipated inaction regret had a significantly positive association with vaccination intention (i.e., the stronger the participants’ anticipated inaction regret, the higher their vaccination intention) [21,32,33,37,39], among which, two reported a stronger association between anticipated inaction regret and vaccination intention than cognitive beliefs [33,39]. However, one of those two studies reported a significantly positive association for males, but there was no significant association for females [33]. Additionally, two intervention studies reported that anticipated inaction regret had a significantly positive effect on vaccination intention [28,40].

Four studies with either cross-sectional or longitudinal designs examined the association between anticipated inaction regret and self-reported vaccination behavior. All four studies reported that anticipated inaction regret had a significantly positive association with self-reported vaccination behavior (i.e., the stronger the participants’ anticipated inaction regret, the more likely they were to be vaccinated) [24,27,31,35]. Among which, two reported a stronger association between anticipated inaction regret and self-reported vaccination behavior than cognitive beliefs [31,35]. Four other cross-sectional studies reported that anticipated inaction regret had a significantly positive association with vaccination willingness and acceptability [22,23,25,30], among which, three reported a stronger association between anticipated inaction regret and the outcomes than cognitive beliefs [22,23,25].

Four studies examined anticipated inaction regret and worry [29], anticipated inaction worry and anxiety reduction [32,39], and anticipated positive and negative affect [36] and evaluated their associations with self-reported vaccination behavior [29] and vaccination intention [32,36,39]. Three of the four studies reported that anticipated affect had a significantly positive association with the outcomes [29,32,36], among which, two reported a stronger association between anticipated affect and the outcomes than cognitive beliefs [29,32]. However, one study reported that inaction worry was not significantly associated with vaccination intention [39].

Seven studies adopted a cross-sectional or longitudinal design to examine the association between anticipated action (i.e., vaccination) regret and self-reported vaccination behavior [20,24,27,31], intention [21], and willingness [25,30]. Four of these studies reported that anticipated action regret had a significantly negative association with the outcomes (i.e., the stronger the participants’ anticipated action regret, the less likely they were to be vaccinated) [20,25,30,31], among which, one reported a stronger association between anticipated action regret and vaccination intention than cognitive beliefs [20]. However, one of those four studies reported a significant positive association for parents, but no significant association was reported for sons [30]. On the contrary, three of the seven studies showed no significant association between anticipated action regret and outcomes [21,24,27].

## 4. Discussion

### 4.1. The Current State and Gaps of the Literature

The present study reviewed articles that examined the types of anticipated affects that would encourage and discourage HPV vaccination. In terms of the types of anticipated affects, the present review found that the majority of the studies (19 studies) examined anticipated inaction regret (i.e., not being vaccinated). This result is consistent with those of previous studies on anticipated affects [11]. The second-highest number of studies (nine studies) examined anticipated action regret (i.e., being vaccinated). Two types of anticipated action regret were reported in those studies: anticipated regret that vaccine-adverse events may occur [21,25,26,27,30,31,38] and anticipated regret that daughters may become sexually active [20,24]. Thus, most previous studies have focused on anticipated inaction regret that may encourage HPV vaccination and anticipated action regret that may discourage HPV vaccination. Previous studies on anticipated affect have focused on inaction regret and, to a lesser extent, anticipated inaction guilt [11]. However, the current review found that no study on HPV vaccination examined anticipated inaction guilt. Regret arises when an individual’s action or inaction results in a negative outcome for oneself. By contrast, guilt arises when an individual’s action or inaction results in a negative outcome for another person [41]. As a result, if their daughters develop cervical cancer due to their inability to receive the HPV vaccination, their parents will feel guilty for not allowing them to receive the vaccine. Furthermore, if their daughters experience vaccine-related adverse events after receiving an HPV vaccination, their parents will feel guilty about vaccinating their daughters. Therefore, future studies should examine the effects of anticipated inaction and action guilt on HPV vaccination. Furthermore, future studies should examine anticipated guilt in terms of gain- and loss-framed appeals, which might differ in the likelihood of evoking parental anticipated guilt. A gain-framed message such as “if you have your daughter receive HPV vaccine, your daughter will be protected against disease” may be less likely to evoke anticipated guilt than a loss-framed message, “if you do not have your daughter receive HPV vaccine, your daughter will not be protected against disease,” because the latter message specifically points to situations that may evoke guilt in a way that the former message does not [42]. Thus, anticipated affect can be a potential moderator and mediator in gain- and loss-framed appeals [42].

The present review found that most of the quantitative studies (thirteen studies) adopted a cross-sectional design, only three studies adopted a longitudinal design, and only three were intervention studies [9]. However, participants’ anticipated affects can be manipulated by simply asking them about it [24]. This question-behavior effect was assessed in previous intervention studies examining health behaviors, such as condom use [43] and cervical cancer screening [14]; namely, participants were randomly allocated to complete a questionnaire with anticipated affect questions or without anticipated affect questions. Participants’ anticipated affect can also be manipulated by intervention messages [44]. Future studies should examine the anticipated affect toward HPV vaccination using experimental designs to accumulate stronger evidence.

Among the participants, young females and parents of daughters were included in ten studies; adult males and parents of sons in six studies; and both females and males in five studies. In the present review, it remained unclear whether the effects of the anticipated affect on HPV vaccination differed between parents and children or between males and females; this should be examined in future studies. The present review found that nine studies examined vaccination intentions, whereas six studies examined self-reported vaccination behavior. The gap between intention and actual behavior should also be noted [45]. Additionally, the gap between self-reported vaccination behavior and actual vaccination should be determined because self-reported behavior can be influenced by social desirability [46] and recall biases [47]. Hence, future studies should examine the actual HPV vaccination as an outcome.

### 4.2. Effect of Anticipated Affect and Recommendations for Future Studies and Practices

The present review investigated the affective impact by comparing it with the cognitive impact of HPV vaccination. Most of the included studies reported that anticipated inaction regret (i.e., not being vaccinated) had a significantly positive association with HPV vaccination outcomes, such as vaccination behavior, intention, willingness, and acceptability, among which, seven had a significantly positive and stronger association with vaccination outcomes than cognitive beliefs, such as vaccine effectiveness and safety, and perceived susceptibility and severity [22,23,25,31,33,35,39]. Additionally, two studies reported that anticipated inaction worry had a significantly positive and stronger association with vaccination outcomes than cognitive beliefs [29,32]. Previous longitudinal studies reported that anticipated inaction regret is a predictor of influenza vaccination [48,49,50,51] and childhood vaccination behaviors [52,53]. Despite the observational nature of these studies, the results of the present review indicate that the stronger people have anticipated inaction negative affects, such as regret and worry, the more likely they are to receive or have their children receive HPV vaccination. However, as mentioned earlier, more intervention studies are needed to determine whether communication strategies targeting anticipated inaction negative affects can encourage HPV vaccination.

Five studies reported that the more participants had anticipated action regrets (i.e., being vaccinated) due to adverse reactions, the lower their intention to receive or have their children receive HPV vaccination [20,25,30,31,38]. As mentioned earlier, anticipated action regret includes concerns about vaccine adverse effects and the vaccine’s effect on sexual behavior. These concerns are barriers to receiving HPV vaccination [54] and can lead to vaccine hesitancy, defined as “delay in acceptance or refusal of vaccines despite availability of vaccination service,” which is a problem that is attracting growing attention and concern [55]. Concerns about vaccine adverse effects, in particular, can influence confidence (i.e., distrust of the vaccine or provider), which, according to the World Health Organization’s Strategic Advisory Group of Experts on Vaccine Hesitancy, is one of the factors of vaccine hesitancy [56]. Therefore, anticipated action regret may discourage HPV vaccination, as indicated in the five studies included in the present review. However, three studies in the present review reported that anticipated action regret was not significantly associated with vaccination outcomes [21,24,27]. Hence, more studies are needed to determine whether anticipated action regret is associated with HPV vaccination uptake and whether interventions that reduce anticipated action regret encourage HPV vaccination.

This scoping review had several limitations. Despite using a comprehensive search strategy, we could not completely rule out the possibility of the incomplete retrieval of relevant studies. The present review might also have missed important literature published in languages other than English. Additionally, studies of HPV vaccine promotion using a narrative or normative approach were not included in this review because this review focused on studies of anticipated affect. Future reviews should focus on studies of HPV vaccine promotion using a narrative or normative approach because it can be an effective strategy to encourage HPV vaccination [57]. Furthermore, as this was a scoping review, we did not conduct formal quality and risk of bias assessments or use meta-analysis techniques. The limitations associated with the qualitative synthesis of the evidence should also be acknowledged.

## 5. Conclusions

The majority of the studies included in this review found a significant and positive association between anticipated inaction negative affect and HPV vaccination outcomes; that is, the stronger the participants’ anticipated inaction negative affect, the more likely they were to receive or have their children receive HPV vaccination. Furthermore, some studies showed that anticipated inaction negative affect was associated with HPV vaccination outcomes as much as or more than cognitive beliefs. Thus, the present review indicated that anticipated affects were associated with HPV vaccination outcomes and may influence people’s vaccination decisions. Messages targeting the anticipated affect may be equally or more effective in encouraging HPV vaccination than those targeting cognitive beliefs. Government agencies and health professionals can use messages that can target the anticipated inaction negative affect, such as regret (e.g., if you do not receive HPV vaccination, you will feel regretful when you develop HPV-related diseases in the future. Choose to receive HPV vaccination to avoid feelings of regret) as well as messages based on cognitive-behavioral models (such as conveying perceived susceptibility and severity, and vaccine effectiveness and safety). However, most of the included studies in the present review adopted a cross-sectional design with vaccination intention and willingness as outcomes. Hence, future studies should examine the influence of anticipated affects on the uptake of HPV vaccines using experimental designs to accumulate stronger evidence.

## Figures and Tables

**Table 1:** Summary of included studies in this review.

Author (year)	Country	Anticipated Affect	Design	Participants (n)	Primary Outcome	Key Findings ^a^
Ziarnowski et al. (2009) [20]	US	Inaction regret, action regret	Cross-sectional	Parents of adolescent girls (886)	Behavior(self-reported)	Action regret (OR = 0.60) and perceived likelihood of cervical cancer (OR = 0.27) was significantly associated with behavior. Inaction regret (β = 0.45), action regret (β = −0.22), and perceived likelihood of cervical cancer (β = 0.19) were significantly associated with intention. Perceived severity of cervical cancer was not significantly associated with both behavior and intention.
Morison et al. (2010) [21]	UK	Inaction regret, action regret	Cross-sectional	Parents of adolescent girls (245)	Intention	Perceived vaccine efficacy (β = 0.38), attitude (β = 0.26), and inaction regret (β = 0.16) were significantly associated with intention. Action regret was not significantly associated with intention.
Reiter et al. (2010) [22]	US	Inaction regret	Cross-sectional	Gay (236) and bisexual (70) adults	Acceptability	Inaction regret (OR = 2.39), effectiveness of vaccine (OR = 1.97), and severity of disease (OR = 1.92) were significantly associated with acceptability. Concern about catching disease and likelihood of catching disease were not significantly associated with acceptability.
Reiter et al. (2010) [23]	US	Inaction regret	Cross-sectional	Heterosexual men (297)	Acceptability	Inaction regret (OR = 2.01), effectiveness of vaccine (OR = 1.86), and likelihood of catching disease (OR = 1.80) were significantly associated with acceptability. Concern about catching disease was not significantly associated with acceptability.
Brewer et al. (2011) [24]	US	Inaction regret, action regret	Longitudinal	Parents of adolescent girls (650)	Behavior (self-reported)	Intention (RR = 2.04) was the strongest significant predictor of behavior, followed by inaction regret (RR = 1.85), and perceived barrier (RR = 0.57). Action regret, perceived harm of vaccine, and uncertainty about vaccine were not significant predictors.
Reiter et al. (2011) [25]	US	Inaction regret, action regret	Cross-sectional	Parents of adolescent males (547) and their sons (421)	Willingness	For parents, inaction regret (β = 0.32), effectiveness of vaccine (β = 0.20), action regret (β = −0.14), harm of vaccine (β = −0.12), and likelihood of catching disease (β = 0.07) were significantly associated with willingness. For sons, peer acceptance of vaccine (β = 0.39), action regret (β = −0.26), inaction regret (β = 0.22), and likelihood of catching disease (β = 0.16) were significantly associated with willingness.
Craciun et al. (2012) [26]	Romania	Action regret	Qualitative	Mothers of adolescent girls (25)	Not applicable	Mothers perceived the HPV vaccine as risky. Their risk perception seemed to link with anticipated action regret about the possible negative effects of vaccination.
McRee et al. (2014) [27]	US	Inaction regret, action regret	Cross-sectional	Lesbian and bisexual young females (543)	Behavior (self-reported)	Social norms (OR = 1.72), inaction regret (OR = 1.69), harm of vaccine (OR = 0.59), and barriers to vaccination (OR = 0.24) were significantly associated with behavior. Action regret, effectiveness of vaccine, worry about catching disease, risk of catching disease were not associated with behavior.
Cox et al. (2014) [28]	US	Inaction regret	Intervention (2 × 2 between-subjects factorial design; asked or not asked anticipated regret questions × text only or graphical presentation of HPV risk)	Mothers of adolescent girls (320)	Intention	Anticipated inaction regret questions positively influenced intention only among mothers exposed to the graphical presentation of HPV-related statistics.
Hofman et al. (2014) [29]	Netherlands	Inaction regret and worry	Longitudinal	Parents of adolescent girls (793)	Behavior (self-reported)	Inaction regret and worry significantly predicted behavior (OR = 1.43). Normative belief, knowledge, susceptibility, and severity were not significant predictors.
Moss et al. (2015) [30]	US	Inaction regret, action regret	Cross-sectional	Parents of adolescent males (412) and their sons (412)	Willingness	For parents, perceived importance of son’s partner being protected (OR = 2.85), inaction regret (OR = 1.72), and action regret (OR = 0.70) were significantly associated with willingness. For sons, perceived importance of a partner being protected (OR = 1.95), likelihood of infection (OR = 1.86), inaction regret (OR = 1.51), and pain from vaccination (OR = 0.55) were significantly associated with willingness.
Krawczyk et al. (2015) [31]	Canada	Inaction regret, action regret	Cross-sectional	Parents of adolescent girls (774)	Behavior (self-reported)	Inaction regret (OR = 1.69), social norms (OR = 1.65), positive attitudes (OR = 1.13), negative attitudes (OR = 0.89), and action regret (OR = 0.61) were significantly associated with behavior.
Wang et al. (2015) [32]	Hong Kong	Inaction regret, inaction worry, anxiety reduction by vaccination	Cross-sectional	Parents of adolescent girls (368)	Intention	Inaction worry (β = 0.23), anxiety reduction (β = 0.19), proneness to peer influence (β = 0.17), susceptibility (β = 0.17), inaction regret (β = 0.14), and descriptive norms (β = 0.13) were significantly associated with intention.
Christy et al. (2016) [33]	US	Inaction regret	Cross-sectional	Undergraduate students (233)	Intention	For men, inaction regret was significantly associated with intention (β = 0.29). Severity of disease, benefit of vaccine, and risk of disease were not significantly associated with intention. For women, benefit of vaccine was significantly associated with intention (β = 0.44). Severity of disease, risk of disease, and inaction regret were not significantly associated with intention.
Pitts et al. (2017) [34]	US	Inaction regret	Qualitative	College-aged males (84)	Not applicable	Participants perceived that vaccination would offer “peace of mind” and taking a preventative step now could relieve potential regret in the future.
Wang et al. (2017) [35]	Hong Kong	Inaction regret	Longitudinal	Parents of adolescent girls (979)	Behavior (self-reported)	Inaction regret (β = 0.32) was the strongest significant predictor of intention, followed by descriptive norms (β = 0.28), benefits of vaccination (β = 0.17), attitudes (β = 0.09); intention, in turn, significantly predicted behavior (β = 0.0.31).
Murray (2019) [36]	US	Anticipated positive and negative affect	Cross-sectional	Young adults (219)	Intention	Instrumental attitudes (β = 0.42), anticipated affective reactions (β = 0.26), and subjective norms (β = 0.23) were significantly associated with intention.
Caso et al. (2019) [37]	Italy	Inaction regret	Cross-sectional	Mothers of adolescent boys (333)	Intention	Subjective norm (β = 0.31), inaction regret (β = 0.26), trust in institution (β = 0.23), behavioral control (β = 0.11), and attitude (β = 0.04) were significantly associated with intention.
Kim (2020) [38]	Korea	Inaction regret, action regret	Intervention (between-subject design)	Undergraduate students (222)	Intention	The didactic message evoked greater anticipated inaction regret than the narrative message. The didactic message (vs. narrative message) significantly increased anticipated inaction regrets (β = −0.410), which in turn significantly increased attitudes (β = 0.197), intention regarding free shots (β = 0.386), and intention regarding paid shots (β = 0.352). The indirect effect via anticipated action regret was not significant, although action regret significantly predicted intention.
Penta et al. (2020) [39]	Romania	Inaction regret, inaction worry	Cross-sectional	Young adults (401)	Intention	Inaction regret (β = 0.38), vaccine safety (β = 0.22), susceptibility (β = 0.16), and vaccine effectiveness (β = 0.13) were significantly associated with intention. Inaction worry was not significantly associated with intention.
Kim et al. (2022) [40]	US	Inaction regret	Intervention (between-subject design)	Young adults (347)	Intention	Loss-famed message led to a significantly stronger inaction regret (M = 4.63, SD = 1.40) than gain- framed message (M = 4.29, SD = 1.73). Participants in the future-thinking condition experienced significantly stronger level of inaction regret (M = 4.72, SD = 1.51) than no-thinking condition (M = 4.38, SD = 1.70) and past-thinking condition (M = 4.35, SD = 1.44).

^a^ Key findings described psychosocial variables; variables that cannot be changed, such as sociodemographic and past vaccination behaviors were not described in the table.

**Table 2:** Summary of key findings of quantitative studies based on the type of anticipated affect and primary outcome *.

	Inaction Regret	Action Regret	Inaction Regret and Worry	Inaction Worry	Anxiety Reduction	Positive and Negative Affect
Behavior (self-reported)	L〇 [24], C〇 [27], C◎ [31], L◎ [35]	C⊗ [20], L● [24], C● [27], C⊖ [31]	L◎ [29]			
Intention	C〇 [21], I〇 [28], C〇 [32], C◎ (for men) [33], C● (for women) [33], C〇 [37], I〇 [38], C◎ [39], I〇 [40]	C● [21], I〇 [38]		C◎ [32], C● [39]	C◎ [32]	C〇 [36]
Willingness	C◎ (for parents) [25], C◯ (for sons) [25], C〇 (for both parents and sons) [30]	C⊖ (for both parents and sons) [25], C⊖ (for parents) [30], C● (for sons) [30]				
Acceptability	C◎ [22], C◎ [23]					

* When multiple outcomes were measured, the primary outcomes (e.g., behavior) were presented in this Table. C: A cross-sectional study. L: A longitudinal study. I: An intervention study. ◎: The variable of anticipated affect was significantly, positively, and more strongly associated with the outcome than with cognitive variables. ⊗: The variable of anticipated affect was significantly, negatively, and more strongly associated with the outcome than with cognitive variables. 〇: The variable of anticipated affect was significantly and positively associated with the outcome or significantly and positively influenced the outcome. ⊖: The variable of anticipated affect was significantly and negatively associated with the outcome. ●: The variable of anticipated affect was not significantly associated with the outcome or did not significantly influence the outcome.

## Data Availability

The datasets generated and analyzed during the current study are available from the corresponding author on reasonable request.

## Appendix A

Preferred Reporting Items for Systematic reviews and Meta-Analyses extension for Scoping Reviews (PRISMA-ScR) Checklist.

**Section**	**Item**	**Prisma-Scr Checklist Item**	**Reported on Page #**
Title			
Title	1	Identify the report as a scoping review.	1
Abstract			
Structured summary	2	Provide a structured summary that includes (as applicable): background, objectives, eligibility criteria, sources of evidence, charting methods, results, and conclusions that relate to the review questions and objectives.	1
Introduction			
Rationale	3	Describe the rationale for the review in the context of what is already known. Explain why the review questions/objectives lend themselves to a scoping review approach.	2
Objectives	4	Provide an explicit statement of the questions and objectives being addressed with reference to their key elements (e.g., population or participants, concepts, and context) or other relevant key elements used to conceptualize the review questions and/or objectives.	2
Methods			
Protocol and registration	5	Indicate whether a review protocol exists; state if and where it can be accessed (e.g., a Web address); and if available, provide registration information, including the registration number.	Not applicable
Eligibility criteria	6	Specify characteristics of the sources of evidence used as eligibility criteria (e.g., years considered, language, and publication status), and provide a rationale.	3
Information sources	7	Describe all information sources in the search (e.g., databases with dates of coverage and contact with authors to identify additional sources), as well as the date the most recent search was executed.	3
Search	8	Present the full electronic search strategy for at least 1 database, including any limits used, such that it could be repeated.	3
Selection of sources of evidence	9	State the process for selecting sources of evidence (i.e., screening and eligibility) included in the scoping review.	4
Data charting process	10	Describe the methods of charting data from the included sources of evidence (e.g., calibrated forms or forms that have been tested by the team before their use, and whether data charting was done independently or in duplicate) and any processes for obtaining and confirming data from investigators.	4-5
Data items	11	List and define all variables for which data were sought and any assumptions and simplifications made.	4−5
Critical appraisal of individual sources of evidence	12	If done, provide a rationale for conducting a critical appraisal of included sources of evidence; describe the methods used and how this information was used in any data synthesis (if appropriate).	Not applicable
Synthesis of results	13	Describe the methods of handling and summarizing the data that were charted.	4−5
Results			
Selection of sources of evidence	14	Give numbers of sources of evidence screened, assessed for eligibility, and included in the review, with reasons for exclusions at each stage, ideally using a flow diagram.	4−5
Characteristics of sources of evidence	15	For each source of evidence, present characteristics for which data were charted and provide the citations.	5−10
Critical appraisal within sources of evidence	16	If done, present data on critical appraisal of included sources of evidence (see item 12).	Not applicable
Results of individual sources of evidence	17	For each included source of evidence, present the relevant data that were charted that relate to the review questions and objectives.	5−10
Synthesis of results	18	Summarize and/or present the charting results as they relate to the review questions and objectives.	5−10
Discussion			
Summary of evidence	19	Summarize the main results (including an overview of concepts, themes, and types of evidence available), link to the review questions and objectives, and consider the relevance to key groups.	11−12
Limitations	20	Discuss the limitations of the scoping review process.	12
Conclusions	21	Provide a general interpretation of the results with respect to the review questions and objectives, as well as potential implications and/or next steps.	13
Funding			
Funding	22	Describe sources of funding for the included sources of evidence, as well as sources of funding for the scoping review. Describe the role of the funders of the scoping review.	13
